# Toward a cognitive framework of expert singing-voice evaluation in bel canto pedagogy: a longitudinal exploratory study

**DOI:** 10.3389/fpsyg.2026.1896993

**Published:** 2026-10-07

**Authors:** Kyoko Ikeda, Michiko Yamaguchi, Akemi Obata, Tomohiro Tani, Yasuki Yamashita, Taisei Terauchi, Takashi Nagai, Yuko Mesuda, Mizue Kayama

**Affiliations:** 1 Faculty of Engineering, Shinshu University, Nagano, Japan; 2 Musashino Academia Musicae, Tokyo, Japan; 3 Nagano Prefectural Institute of Technology, Ueda, Japan; 4 Institute of Technologists, Gyoda, Japan; 5 National Institute of Technology, Nagano College, Nagano, Japan

**Keywords:** acoustic analysis, bel canto pedagogy, evaluative language, expert judgment, Polanyi, singing-voice evaluation, tacit knowledge, Vygotsky

## Abstract

Classical singing pedagogy relies on expert judgments that integrate auditory-perceptual cues, embodied pedagogical knowledge, and specialized evaluative language. This exploratory, longitudinal study proposes a cognitive framework, centered on Polanyi’s concept of tacit knowledge, for describing how experienced bel canto pedagogues perceive singing-voice development from a brief excerpt. Twenty female soprano students (7 music-education, 13 vocal-performance majors) sang the word ‘tanto’ from Caro mio ben at university entry (Z1) and after 2 years of instruction (Z2). Four professional singers and voice pedagogues, blind to institutional affiliation, rated perceived development on a 0–2 scale and selected evaluative terms from a 27-item Italian pedagogical vocabulary; median ratings, rather than the mean of an even number of raters, served as the primary outcome. Acoustic analysis proceeded in two exploratory, descriptive stages. First, the singer’s-formant ratio (SFR) and a Q-value index were compared with a pedagogical reference recording (M2025) using path-length-normalized dynamic time warping. Movement toward M2025 did not correspond consistently with expert ratings: several students whose SFR/Q-value profiles moved toward M2025 received low or zero median ratings (e.g., E06, E07, V07), whereas six students whose profiles moved away from M2025 on both indices received high median ratings of 1.5–2 (E02, E05, V01, V02, V03, V06). Second, changes in alpha ratio, H1-H2, spectral centroid, smoothed cepstral peak prominence (CPPS), and singing power ratio (SPR) were examined descriptively alongside participants’ evaluative terms, without regression or machine-learning modeling, given the small, non-random sample. Case-level analysis indicates that experts attended to qualitatively different cues depending on what they heard in a given voice—e.g., basic, mouth-opening- and resonance-related terms (S07, R02) versus depth- and vibrato-related terms (R10, S09)—independent of institutional cohort. We propose a Polanyi-centered interpretive framework in which candidate acoustic correlates, the 27 evaluative terms (understood, after Vygotsky, as mediating cultural-linguistic tools), and Dreyfus-and-Dreyfus-informed developmental sensitivity jointly, but only partially, illuminate the focal judgment that pedagogically meaningful development has occurred. The framework is hypothesis-generating rather than confirmatory; no predictive or inferential claims are made about the acoustic determinants of expert judgment.

## 1 Introduction

In classical singing education, experienced teachers often infer changes in phonation, resonance, and breath management from very brief excerpts of a learner’s singing. Such judgments are commonly expressed through specialized, metaphorical, and pedagogically situated language (Crisosto-Alarcón et al., 2025), such as ‘the voice became brighter,’ ‘the resonance improved,’ or ‘the tone is less breathy.’ It remains unclear how shareable this evaluative knowledge is, what perceptual information it draws on, and how it might be partially, even if incompletely, described in relation to acoustic measurement.

Polanyi (1966) argued that ‘we can know more than we can tell,’ introducing tacit knowledge as a form of knowing that is not fully reducible to explicit verbal rules. In Polanyi’s framework, expert judgment integrates subsidiary particulars—cues that are not individually the object of attention—into a focal awareness. The present study treats Polanyi’s distinction as its theoretical center: rather than asking which acoustic variable predicts an expert score, it asks what candidate subsidiary particulars might be integrated into the focal judgment that a learner’s singing has developed in a pedagogically meaningful way, and how far this process can be partially, provisionally illuminated through coordinated acoustic and linguistic analysis.

The five-stage skill-acquisition model of Dreyfus and Dreyfus (1986) is used as an interactive lens rather than as a categorical claim about the two student cohorts. Novices rely on context-free rules; experts grasp situations holistically. In the present analysis, developmental stage is treated as a property that may vary from voice to voice, and even within a single participant’s recording, rather than as a fixed label attached to institutional group membership (music-education cohort versus vocal-performance cohort). Because the two cohorts differed in prior selection, curriculum, and instructional exposure (Table 1), any between-cohort acoustic or terminological differences are confounded with these institutional factors and are not attributed to developmental stage as such; see Limitations.

**Table 1:** Participant characteristics.

Cohort	N	Sex	Age at Z1	Voice type	Prior specialized training at Z1	Instruction between Z1 and Z2	Approx. individual instruction over 2 years
Music-education course (E01-E07)	7	Female	18–19	Soprano	Little or none	Weekly 90-min group classes (~60 sessions)	~20 h
Vocal performance majors (V01-V13)	13	Female	18–19	Soprano	Entrance-examination preparation	Weekly 60-min individual lessons	~60 h

The two cohorts differ not only in prior training but in institution curriculum teacher selection procedure and total instructional exposure; cohort membership is therefore not treated as equivalent to a Dreyfus-and-Dreyfus developmental stage (Sections 2.2.2 and 4.3 and Limitations). E, music-education cohort; V, vocal-performance cohort.

A substantial body of singing-voice research has examined the acoustic and physiological basis of classical singing. Since the work of Sundberg (1974, 1987, 2001), the concentration of energy in the 2–4 kHz region known as the singer’s formant has been regarded as a central acoustic feature of trained classical singing (Sundberg, 1974, 2001; Bloothooft and Plomp, 1986). Later studies have examined formant-tuning strategies, vocal-tract configuration, harmonic structure, and phonatory mechanisms in professional singing (Sundberg et al., 2013, 2023; Takahashi et al., 2026).

Research on voice quality and timbre has also identified acoustic measures relevant to perceived singing-voice quality. Smoothed cepstral peak prominence (CPPS) has been used as an index of harmonic organization and noise level (Hillenbrand et al., 1994; Fraile and Godino-Llorente, 2014). H1-H2 is commonly interpreted as an acoustic correlate influenced by phonatory configuration, although, uncorrected for formant effects as used here, it also reflects source-filter interaction rather than glottal closure alone (Garellek and Keating, 2011; Vurma, 2017). The alpha ratio indexes the balance between low- and high-frequency energy (Patel et al., 2010; Eyben et al., 2016). The spectral centroid is associated with timbral brightness (Wessel, 1979; Schubert and Wolfe, 2006; Liu et al., 2022), and the singing power ratio (SPR) quantifies the relative strength of the singer’s-formant region (Omori et al., 1996).

Despite this rich acoustic literature, comparatively little research has examined how expert teachers perceive developmental change in individual learners over time, how they may relate auditory impressions to evaluative language, and how their judgments may depend on pedagogical context. Bel canto teaching has historically been transmitted through face-to-face interaction, in which language functions as a practical tool for reorganizing the learner’s bodily action and auditory imagery. Terms such as voce in maschera, appoggio, and sul fiato are not merely labels; they may operate as culturally shared cues that guide attention and motor adjustment. Sundberg (1987) noted that common singing terms may be used differently by different individuals, a methodological challenge for scientific work on singing pedagogy.

Building on Vygotsky's (1978) concept of language as a psychological tool, the present study treats the 27 evaluative terms that language is ‘embedded’ in judgment, but as a theoretically motivated candidate mediating device: a culturally shared vocabulary that may allow experts to select, stabilize, and communicate part of an otherwise holistic auditory impression. This role is argued on theoretical grounds and through qualitative case description, not established through the term-count/rating correlation, which is procedurally confounded (evaluators selected terms only when they perceived some development, so a rating of zero mechanically yields few or no terms; see Sections 2.5.2 and 3.1).

The present study examines these theoretical questions through a single, tightly defined case: the brief fragment ‘tanto’ from the Italian song Caro mio ben, attributed to Tommaso Giordani, which the expert panel identified as pedagogically informative because it contains an E♭5 pitch (approximately 622 Hz), a sustained tone, and the open vowel /a/. The entire word, including the consonant-to-vowel onset and the transition to /o/, was analyzed as a single perceptual unit rather than an isolated steady-state vowel segment, reflecting the view that experts integrate onset, sustain, and release into one judgment of ‘tanto’ as a whole—a further candidate instance of subsidiary particulars integrated into a focal judgment, revisited as a limitation in Section 5 because consonantal and transitional content can influence some of the acoustic measures used here. Around this fragment, the study integrates three kinds of data collected from the same 20 students at two time points, Z1 (university entry) and Z2 (after 2 years of instruction): acoustic features extracted from the singing voice, ordinal expert ratings of perceived longitudinal development, and evaluative terms selected by four professional singers and voice pedagogues from a 27-item Italian vocabulary organized into voice-source, resonance, and respiratory/breath-support categories.

Acoustic analysis proceeded in two exploratory, purely descriptive stages, neither of which is intended to predict or explain expert ratings in an inferential-statistical sense. First, SFR and Q-value measures related to the singer’s-formant region (Yoshida et al., 2020; Yamashita et al., 2024) were compared with the pedagogical reference recording M2025 using path-length-normalized dynamic time warping (DTW; Sakoe and Chiba, 1978), because unnormalized cumulative DTW cost can depend on sequence and warping-path length. Because SFR/Q-value proximity to M2025 did not correspond consistently with expert ratings (Section 3.2), a second stage then examined alpha ratio, H1-H2, spectral centroid, CPPS, and SPR case by case, alongside the evaluative terms associated with individual participants. These two stages, together with the case-level evaluative-language analysis, were designed to address three research questions: RQ1, to what extent, if at all, do SFR and Q-value changes relative to a pedagogical reference model correspond to expert ratings of longitudinal development? RQ2, when SFR/Q-value proximity and expert ratings diverge, what do the five additional acoustic measures and the evaluative terms selected for the same participants suggest about what the experts may have been attending to? RQ3, how might the 27 evaluative terms, considered as Vygotskian mediating tools, and the Dreyfus-and-Dreyfus notion of stage-sensitive judgment, be integrated with Polanyi’s account of tacit knowledge into a single, provisional interpretive framework for expert singing-voice evaluation?

Answering these questions in this exploratory study makes three contributions. First, it proposes a Polanyi-centered cognitive framework, rather than a predictive acoustic model, for describing tacit evaluative knowledge in bel canto pedagogy. Second, it illustrates, through individual case analysis rather than group-level statistical inference, how expert evaluative focus may track the developmental character of what is audible in a given voice rather than a learner’s institutional group. Third, it offers a methodological template—coordinated, descriptive analysis of acoustic change, evaluative language, and case-level context—for studying tacit knowledge in other embodied, language-mediated domains of expertise, while explicitly avoiding statistical claims that a sample of this size and this sampling design cannot support.

## 2 Materials and methods

### 2.1 Study design

This exploratory longitudinal observational study investigated candidate acoustic and linguistic correlates of expert tacit evaluative knowledge in bel canto pedagogy. Vocal data were recorded at two time points: Z1, at university entry, and Z2, after 2 years of instruction (Figure 1). Four professional singers and voice pedagogues auditorily evaluated each student’s perceived development in the target fragment ‘tanto’. Acoustic analysis was conducted in two stages, as described above (Figure 2).

**Figure 1:**
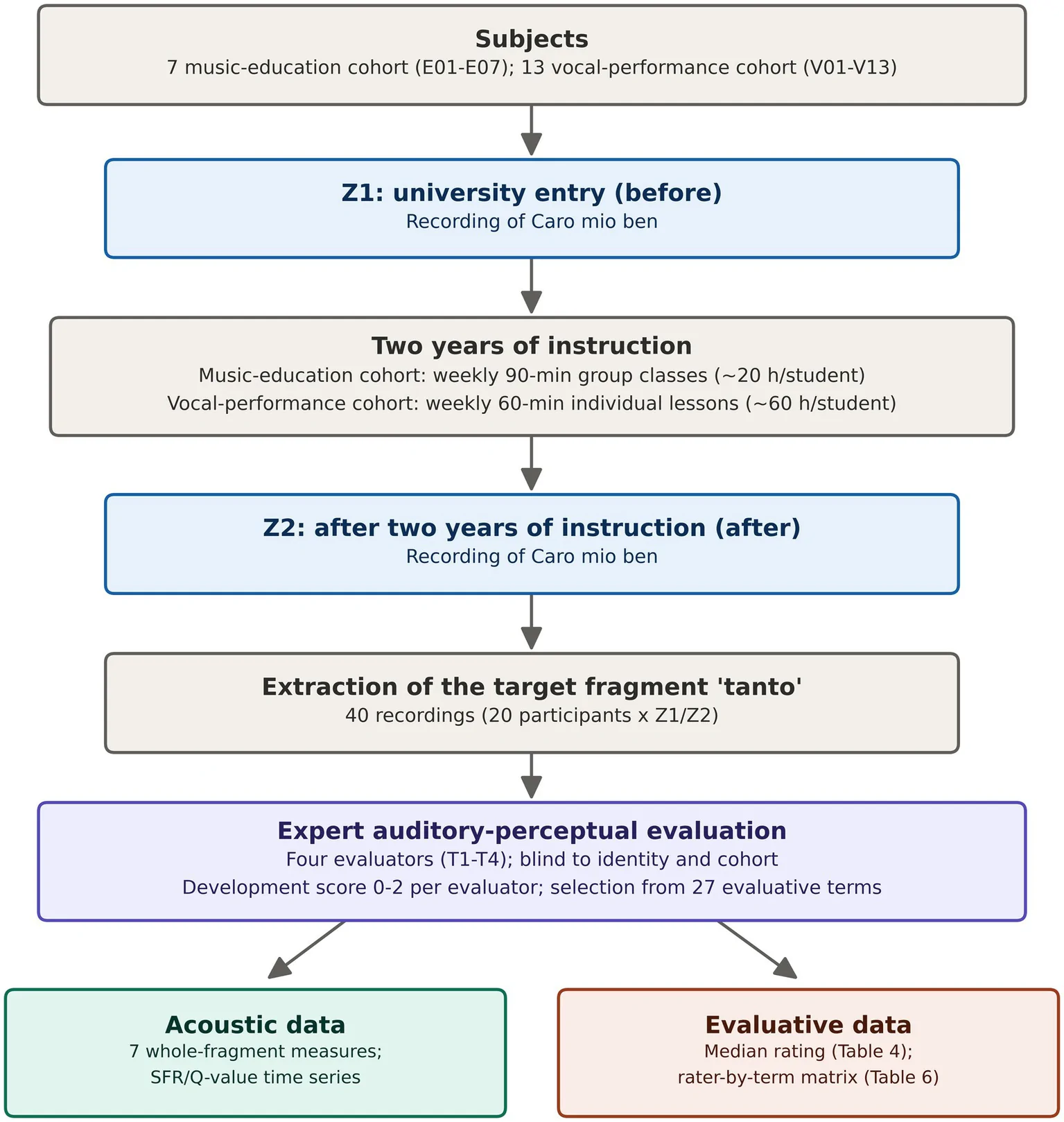
Data acquisition process. Singing data were recorded at Z1 and Z2, the target fragment ‘tanto’ was extracted, and four experts (T1-T4) evaluated each before-after pair using both a 0–2 development score and selection from the 27-item evaluative vocabulary (Table 3). E, music-education cohort; V, vocal-performance cohort; SFR, singer’s-formant ratio.

**Figure 2:**
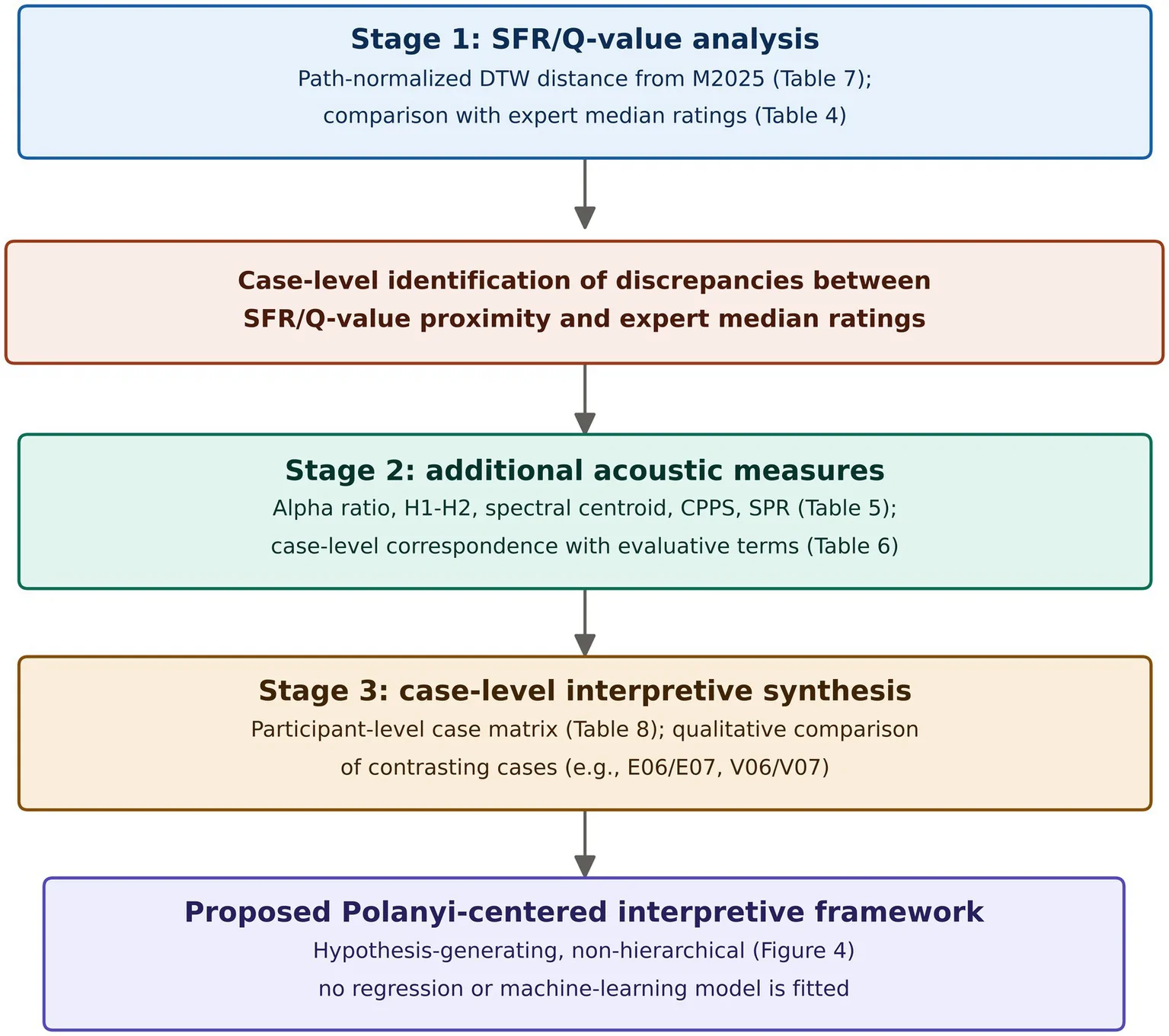
Integrated analysis workflow. Acoustic analysis proceeds from path-length-normalized SFR/Q-value dynamic time warping (DTW) comparison with the pedagogical reference recording M2025 (Stage 1) through case-level discrepancy identification to additional acoustic measures and evaluative-term correspondence (Stage 2), culminating in a case-level interpretive synthesis (Stage 3) that motivates the proposed, non-hierarchical Polanyi-centered framework (Figure 4). CPPS, smoothed cepstral peak prominence; SPR, singing power ratio.

Given the small, non-random, and institutionally confounded sample (*N* = 20; see Section 2.2 and Limitations), no regression model, machine-learning importance ranking, or between-group inferential test is reported in this paper. All quantitative summaries in Sections 3.1–3.3 are descriptive. The interpretive framework proposed in Section 3.4 and Section 4 is presented explicitly as hypothesis-generating.

### 2.2 Participants

#### 2.2.1 Expert evaluators

The evaluation panel consisted of four professional singers and voice pedagogues (T1-T4). At the time of the evaluation experiment, their careers as classical singers were 35, 34, 25, and 43 years, respectively, and their teaching experience was 33, 28, 24, and 37 years. The voice types represented in the panel were two sopranos, one mezzo-soprano, and one baritone; consistent with these voice classifications, three evaluators were female and one was male. Because the same four experts also participated in constructing the evaluative vocabulary (Section 2.5.1) and selecting the pedagogical reference recording M2025 (Section 2.4), the panel is not independent of the materials it subsequently judged; this non-independence, and the associated risk of confirmation bias, is discussed explicitly in Limitations.

#### 2.2.2 Student participants

The student participants were 20 female soprano students: seven students from a university music-education course (E01-E07, referred to here as the music-education cohort) and 13 students majoring in vocal performance at a music university (V01-V13, the vocal-performance cohort). Each participant was recorded at Z1 and Z2. Participant characteristics are summarized in Table 1.

The music-education cohort had received little or no specialized vocal training before Z1. By Z2, they had attended weekly 90-min group classes for 2 years (approximately 60 sessions), with each student receiving approximately 20 min of individual attention per class while also observing peers; this amounted to approximately 20 h of individual instruction per student over 2 years. Teaching materials consisted mainly of Concone’s 50 Lessons and Italian art songs. The vocal-performance cohort had already received vocal training for university entrance examinations before Z1. By Z2, they had continued weekly 60-min individual lessons for 2 years, totaling approximately 60 h of instruction; their repertoire consisted mainly of Italian songs, including classical, Romantic, and modern works.

### 2.3 Audio recording and signal preparation

#### 2.3.1 Recording conditions and preprocessing

Recordings were made with an Olympus LS-P2 IC recorder using its built-in stereo microphone, with the center-microphone setting on, zoom off, low-cut filter off, scene selection off, limiter set to ‘Music’, and recording level set to Auto; automatic gain control was therefore active and was not disabled. Participants sang at a distance of approximately 2 m from the recorder, fixed with a tape measure. Sound-pressure level was not calibrated and amplitude was not normalized before analysis; absolute sound level and inter-participant loudness differences are therefore not interpreted directly. Recordings for the vocal-performance cohort were made in a lesson room of approximately 4.3 m (width) × 5.0 m (depth) × 3.0 m (height); each student’s Z1 and Z2 recordings used the same room, equipment, and settings. For the music-education cohort, room dimensions were not recorded, but all seven students were recorded in the same room with the same equipment and settings at both Z1 and Z2.

All source recordings, including the pedagogical reference recording M2025, were originally acquired with the Olympus LS-P2 as stereo linear PCM WAV files at 44.1 kHz and 16-bit quantization. For analysis, the stereo files were converted to mono in Praat 6.4.27 (Boersma and Weenink, 2025) using Convert > Convert to mono, which averages the left and right channels into a single channel. The resulting mono files were saved as WAV at 44.1 kHz/16-bit linear PCM, with no resampling and no lossy-format intermediate step at any point in the pipeline.

#### 2.3.2 Target fragment extraction

The word ‘tanto’ was extracted from the onset of the initial /t/ to the end of the final /o/ using Praat waveform and spectrogram inspection together with auditory judgment. Extraction was performed by the first author (K. I.) and confirmed by all four expert evaluators. Silence and unvoiced frames at the boundaries of the extracted segment were not algorithmically trimmed beyond this manual boundary selection. The word (rather than an isolated steady-state vowel) was analyzed because the panel judged that the onset, sustain, and vowel transitions jointly carry the perceptual information relevant to ‘tanto’-based development judgments; the acoustic implications of including consonantal and transitional content are discussed in Limitations.

The ‘tanto’ fragment was extracted from the Z1 and Z2 recordings of all 20 students. The resulting 40 samples had a mean duration of 2.21 s, a maximum duration of 3.28 s, a minimum duration of 1.59 s, and a standard deviation of 0.40 s.

#### 2.3.3 Acoustic analysis parameters

Acoustic measures were computed frame-by-frame using 2,048-sample analysis frames (Hann window, FFT size 2,048) at 44.1 kHz. Frame-level parameters specific to each measure (hop size/overlap, LPC order for Q-value peak detection, and any spectral smoothing) are documented in the analysis scripts and README files provided in the data repository (Data availability statement) and summarized in Supplementary Table S1. Frames for which a given measure could not be reliably computed (for example, no detectable spectral peak in the 2.4–4.0 kHz band for the Q-value analysis) were neither imputed, interpolated, nor set to zero; they were excluded from that measure’s per-fragment summary, and the exact representation used in the underlying code (missing value, flag, or omitted row) is documented per measure in the repository.

Acoustic measurements were obtained using Praat (Boersma and Weenink, 2025) and custom Python-based analysis scripts, including use of librosa (McFee et al., 2015).

### 2.4 Pedagogical reference model M2025

The present study did not treat a professional singer’s voice as a single universal ideal target. Instead, it used a single recording, referred to as M2025, as an attainable pedagogical reference point for early bel canto instruction. M2025 is one recording of the word ‘tanto’ by one professional female classical singer; it is not a synthetic, averaged, or mathematically composited signal.

Eight candidate recordings from eight female classical singers (seven sopranos, one mezzo-soprano)—four recordings from 2015, three from 2023, and one from 2025—were considered by the four-member expert panel, who unanimously selected M2025. This selection was made before the panel viewed any of the Z1/Z2 acoustic results, DTW distances, or the present study’s outcomes, which reduces but does not eliminate the risk of confirmation bias, because the same panel also constructed the evaluative vocabulary and rated all 20 participants; this non-independence is acknowledged as a limitation. The selection criterion was a balanced, attainable, non-idiosyncratic phonation rather than a fully formed, highly individual stage voice, on the reasoning that professional voices often carry strong individual artistic characteristics that may not be appropriate targets for beginner instruction. No independent external panel evaluated the pedagogical validity of M2025.

M2025 was recorded and preprocessed in the same manner as the student recordings with respect to file format and stereo-to-mono processing: it was originally recorded as a stereo linear PCM WAV file at 44.1 kHz/16 bit and was converted to mono in Praat 6.4.27 using Convert > Convert to mono (averaging the left and right channels). Table 2 reports the descriptive acoustic profile of M2025 as computed by the present analysis pipeline; these values are presented as a descriptive characterization of the selected reference recording, not as normative physiological thresholds (e.g., a given Q-value or H1-H2 range is not asserted here to indicate ‘pressed’ or ‘healthy’ phonation in general, because the present data do not provide direct evidence for such thresholds).

**Table 2:** Descriptive acoustic profile of the pedagogical reference recording M2025, as computed by the present analysis pipeline.

Parameter	M2025 value (this pipeline)	Descriptive note
Mean SFR	15.45%	Mean proportion of energy in the 2.4–4.0 kHz band relative to 0–4.0 kHz, averaged over valid analysis frames of the ‘tanto’ fragment.
Mean Q-value	31.54	Mean of the frame-level Q-value at the detected spectral-envelope peak in the 2.4–4.0 kHz band, valid frames only.
Spectral centroid	1664.8 Hz	Mean spectral centroid over valid frames.
Alpha ratio	6.06 dB	Mean energy-ratio index (50–1,000 Hz vs. 1,000–5,000 Hz) over valid frames.
SPR	−19.12 dB	Mean singing power ratio (2–4 kHz vs. 0–2 kHz peak difference) over valid frames.
H1-H2 (uncorrected)	6.58 dB	Mean, uncorrected for formant effects; a source-filter-influenced acoustic correlate, not a direct closure measurement.
CPPS	10.58 dB	Mean smoothed cepstral peak prominence over valid frames.

SPR, singer’s formant ratio; SPR, singing power ratio; CPPS, smoothed ceptral peak prominence.

### 2.5 Expert auditory-perceptual evaluation

#### 2.5.1 Selection of evaluative terms

The expert panel first organized expressions used in bel canto pedagogy to describe learner development, producing approximately 300 candidate terms from everyday teaching practice. From these, the panel selected terms considered relatively shareable among experts (i.e., not restricted to a single teacher’s idiosyncratic usage), organized into three categories: voice-source (S01-S10), resonance (R01-R12), and respiratory/breath-support (P01-P05) factors, yielding 27 Italian evaluative expressions (Table 3). Bel canto pedagogy often favors positive formulations indicating a desirable direction rather than directly naming a problem; the vocabulary is predominantly positive in this sense.

**Table 3:** Twenty-seven development-related evaluative expressions used in bel canto pedagogy, with contextual English glosses.

Code	Italian label	Contextual English gloss
S01	non forzare la voce	voice produced with less apparent forcing
S02	voce non ingolata (aperta)	less throaty/constricted, more open-sounding voice
S03	voce morbida	softer, more supple vocal quality
S04	voce non soffiata	less breathy, with reduced audible air leakage
S05	laringe abbassata (si ritiene)	production perceived as involving a lower laryngeal setting
S06	chiusura cordale	sound consistent with firmer vocal-fold closure and a stronger, fuller tone
S07	la bocca si apre bene	more adequate and released mouth opening
S08	intonazione giusta	more accurate intonation
S09	vibrato naturale	more natural, well-formed vibrato
S10	giusta posizione delle vocali	more appropriate vowel placement and articulation
R01	sentire della risonanza	greater awareness/sensation of resonance
R02	voce risonante	more resonant vocal quality
R03	si alza il palato molle	production perceived as involving a raised soft palate
R04	più voluminoso	greater vocal fullness or apparent volume
R05	non è più chiusa dentro	less inward/muffled, more open-sounding voice
R06	innalzamento del punto di risonanza	resonance perceived at a higher position
R07	voce in maschera	more forward, ‘in-the-mask’ resonance
R08	voce che arriva lontano	voice with greater carrying power/projection
R09	diventata più chiara	brighter vocal timbre
R10	voce profonda	greater depth and richness of vocal timbre
R11	voce non nasale	less nasal vocal quality
R12	unificazione delle cinque vocali	more unified resonance across the five vowels
P01	respirazione costo-diaframmatica	breathing perceived as more effectively costo-diaphragmatic
P02	sul fiato	voice carried on a well-coordinated breath flow
P03	buon controllo del respiro	better control and management of the breath
P04	buon appoggio	more stable appoggio and breath-support coordination
P05	buon flusso del fiato	more continuous and well-coordinated breath flow

S, voice-source factor; R, resonance factor; P, respiratory/breath-support factor. Glosses describe pedagogical usage; they are not clinical or anatomical definitions and are not equated *a priori* with any single acoustic measure (see Supplementary Table S2 for term-specific caveats).

Table 3 reports, for each code, the Italian pedagogical label and a contextual English gloss. The gloss is not a literal or clinical translation and does not assert a one-to-one correspondence with any specific acoustic measure or anatomical event; for example, S06 (chiusura cordale) is glossed as ‘sound consistent with firmer vocal-fold closure and a stronger, fuller tone’ because vocal-fold closure is perceptually inferred by the listener from the sound, not directly observed or measured in this study. Term-specific interpretive caveats are given in full in Supplementary Table S2.

#### 2.5.2 Evaluation procedure and blinding

Evaluators were not informed of each participant’s name, institutional cohort (music-education vs. vocal-performance), the acoustic analysis results, or the DTW comparison with M2025. Recordings from both cohorts were interleaved and presented in a randomized order of anonymized participant numbers (1–20). For each participant, the Z1 and Z2 recordings were presented as a pair, in Z1-then-Z2 order, because the evaluation task was explicitly to judge two-year development; evaluators therefore knew the temporal order of the two recordings within a pair, but not the participant’s group or identity (occasion order was not blinded, but group membership was).

Recordings were played back without loudness normalization, using the same computer and a pair of JBL Pebbles loudspeakers for all evaluators; playback level and listening conditions were kept as constant as practicable across evaluators, based on the study records, though this was not instrumentally verified. Evaluators could replay a Z1/Z2 pair as needed before scoring; the number of replays was not logged, and repeated-trial (intra-rater) reliability was not assessed.

Evaluators completed two kinds of judgment for each participant. First, they rated perceived Z1-to-Z2 development on a three-point scale: 0 = no clear longitudinal development perceived, 1 = some development perceived, and 2 = substantial development perceived. A rating of 0 indicates that a given evaluator did not perceive an overall, integrated bel canto development between Z1 and Z2; it does not indicate low vocal quality or the absence of any locally perceptible change; several rating-0 cases co-occur with locally selected evaluative terms (see Section 3.1 and Section 3.3). Second, when an evaluator perceived development, they selected the relevant evaluative term(s) from Table 3; if no development was perceived, no term was selected. Because term selection was conditional on a non-zero rating from the same evaluator on the same trial, term counts and ratings are not procedurally independent, and no correlation between them is reported as evidence of a psychological or cognitive process.

Because an even number (four) of evaluators contributed to each participant’s rating, the median of the four ratings can take the values 0, 0.5, 1, 1.5, or 2 (0.5 and 1.5 arise from averaging the two central values); the median, rather than the mean, is used throughout as the primary summary of perceived development, alongside the individual T1-T4 ratings (Table 4).

**Table 4:** Development ratings (0–2, dimensionless ordinal scale) assigned by each of the four expert evaluators (T1-T4) and the rater-wise median.

Subject	T1	T2	T3	T4	Median
E01	2	1	1	2	1.5
E02	2	1	1	2	1.5
E03	2	0	0	1	0.5
E04	0	1	0	2	0.5
E05	2	2	0	1	1.5
E06	2	0	0	0	0
E07	0	0	0	0	0
V01	2	2	0	2	2
V02	2	2	0	1	1.5
V03	2	2	1	2	2
V04	1	1	0	0	0.5
V05	1	1	0	0	0.5
V06	2	2	1	2	2
V07	0	0	0	0	0
V08	0	0	0	0	0
V09	2	1	0	1	1
V10	2	2	1	1	1.5
V11	1	1	0	1	1
V12	0	0	0	0	0
V13	1	1	1	1	1

Median values of 0.5 and 1.5 arise from averaging the two central values of an even number of raters. A rating of 0 indicates that a given evaluator did not perceive overall, integrated longitudinal development; it does not indicate low vocal quality (Section 2.5.2). E, music-education cohort; V, vocal-performance cohort.

### 2.6 Stage 1: SFR/Q-value path-normalized DTW analysis

The first analytic stage examined the singer’s-formant ratio (SFR) and a Q-value index, following the definitions used by Yoshida et al. (2020) and Yamashita et al. (2024), and compared each participant’s frame-level time series with the corresponding series for M2025 using dynamic time warping (DTW; Sakoe and Chiba, 1978). SFR and Q-value were kept as two separate DTW analyses, rather than combined into a single index, because they represent conceptually and dimensionally different quantities (a band-energy ratio versus the sharpness of a spectral-envelope peak) and combining them would require an arbitrary weighting.

SFR was defined as the ratio of energy in the 2.4–4.0 kHz band to the total energy below 4.0 kHz in each analysis frame. The Q-value at a detected spectral-envelope peak in the 2.4–4.0 kHz range was defined as the peak’s center frequency divided by its −3 dB bandwidth; the maximum Q-value within a frame was used. Frames without a detectable peak in this band were excluded from that frame’s Q-value series rather than imputed (Section 2.3.3).

Because fragment duration varied across recordings (Section 2.3.2), raw DTW cumulative cost is not directly comparable across participants: an unnormalized cumulative cost can depend on the length of the optimal warping path itself, independent of how similar the two series are. This analysis therefore reports a path-length-normalized DTW distance, computed as the cumulative absolute local cost along the optimal warping path divided by the length (number of steps) of that path. The local cost between two points was the absolute difference of their values; the permitted step directions were diagonal, vertical, and horizontal, with no additional global window constraint. No smoothing was applied to the series used for the DTW computation itself (a Savitzky–Golay filter followed by PCHIP interpolation was used only for the display curves in Figure 3, not for the underlying distance calculation).

**Figure 3:**
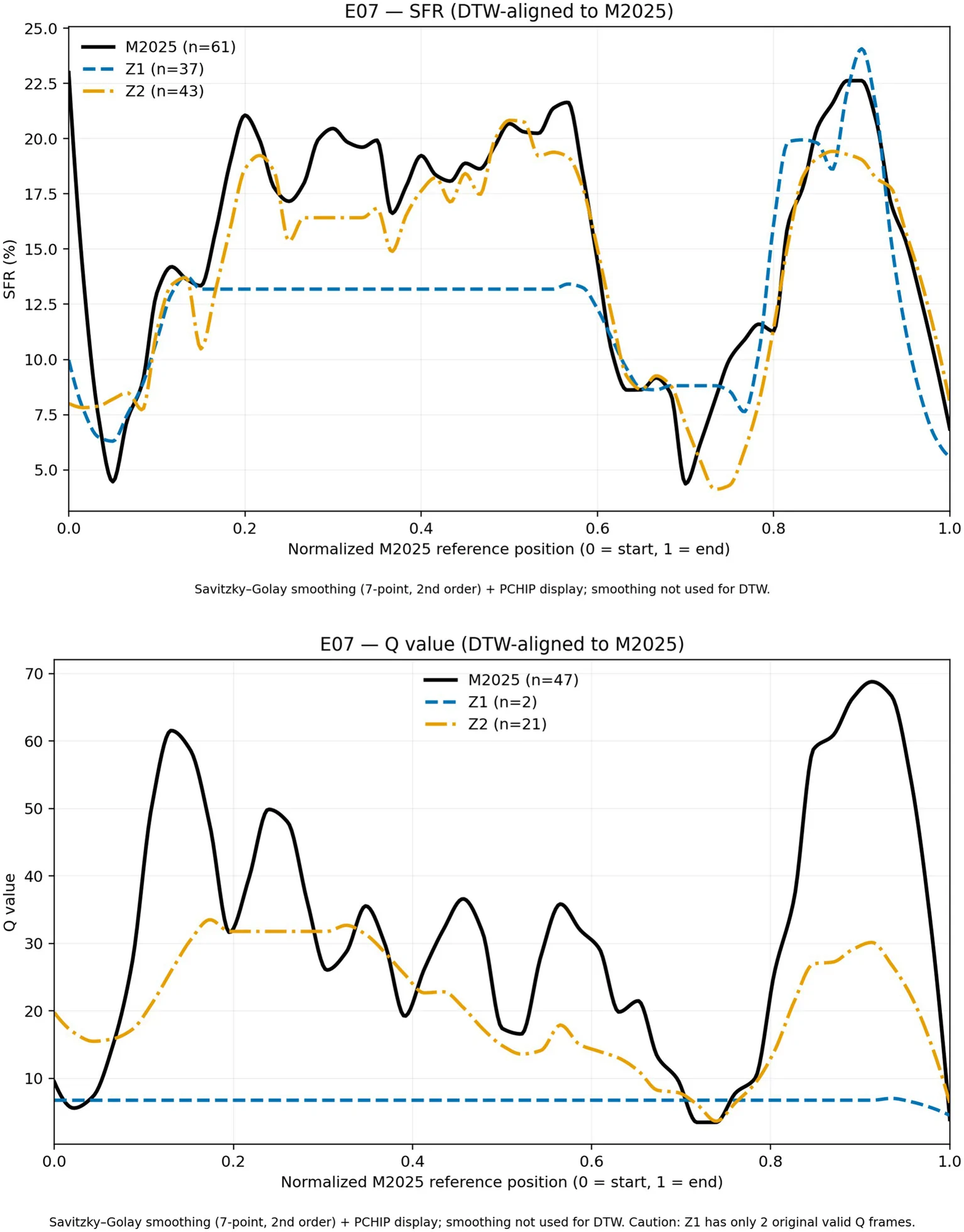
SFR (top) and Q-value (bottom) time series for M2025 (black solid), Z1 (blue dashed), and Z2 (orange dash-dot) for participant E07, DTW-aligned to the M2025 reference and shown on a normalized M2025 reference-position axis (0 = start, 1 = end; not physical time). Curves are Savitzky–Golay-smoothed (7-point, second order) with PCHIP display interpolation for visualization only; smoothing was not used in the underlying DTW distance calculation (Section 2.6). Both SFR and Q-value moved toward M2025 from Z1 to Z2 for E07 (Table 7), yet E07 received a median expert rating of 0 (Table 4), illustrating the central discrepancy examined in Section 3.2. E07’s Z1 series has only 2 valid Q-value frames, shown as a caution in the panel. DTW, dynamic time warping; SFR, singer’s-formant ratio.

For each participant X, a normalized DTW distance to M2025 was computed separately at Z1 and at Z2, for SFR and for Q-value. The primary summary reported here is Δproximity = DTWnorm(Z1) - DTWnorm(Z2): a positive value indicates that the Z2 recording was closer to M2025 than the Z1 recording (movement toward M2025, ‘approach’); a negative value indicates movement away from M2025 (‘divergence’).

Because repeated recordings were not obtained at a single time point, no formal test of whether an observed change exceeds measurement variability is available; approach/divergence is reported descriptively for each participant, without a significance threshold. DTW was used only to compute a numerical correspondence between two time series of different lengths; it does not alter, stretch, or compress the underlying audio recordings, and it does not change the singers’ actual timing or pitch.

### 2.7 Stage 2: additional acoustic measures

Because SFR/Q-value proximity to M2025 did not correspond consistently with expert ratings (Section 3.2), five additional whole-fragment acoustic measures were computed for Z1 and Z2 for descriptive, case-level comparison with the evaluative terms selected by the experts (Table 5).

**Table 5:** Whole-fragment (frame-averaged) acoustic measures at Z1 and Z2 for all participants and for M2025.

Subject	Occasion	Alpha ratio (dB)	CPPS (dB)	H1-H2 (dB, uncorr.)	Mean SFR (%)	SPR (dB)	Spectral centroid (Hz)	Mean Q-value (dimensionless)
M2025	REF	6.06	10.58	6.58	15.45	−19.12	1664.8	31.54
E01	Z1	8.32	10.32	2.94	14.29	−21.47	2246.6	9.74
E01	Z2	10.33	9.95	7.89	15.42	−20.53	2084.9	16.18
E02	Z1	11.72	10.57	5.83	11.12	−23.00	1817.2	13.27
E02	Z2	−2.33	9.25	−3.90	9.35	−24.11	1460.3	16.87
E03	Z1	5.83	7.15	2.41	5.08	−31.94	1777.9	—
E03	Z2	4.60	8.34	0.36	10.53	−25.24	2604.8	5.33
E04	Z1	6.87	8.58	4.79	8.26	−28.27	1786.2	7.93
E04	Z2	6.89	9.18	9.88	12.18	−23.80	2418.1	12.84
E05	Z1	1.82	8.71	−1.60	8.70	−27.38	1487.0	27.54
E05	Z2	−3.72	9.82	6.93	18.03	−20.77	2021.5	19.95
E06	Z1	6.19	6.82	4.73	4.27	−33.20	1343.7	5.70
E06	Z2	2.46	8.69	−0.38	13.14	−22.71	2313.6	9.97
E07	Z1	−1.07	7.44	−5.33	8.27	−26.63	1955.4	5.29
E07	Z2	−4.83	8.44	−7.76	12.39	−23.13	2176.2	13.62
V01	Z1	5.77	8.88	5.44	8.14	−28.06	1536.8	14.27
V01	Z2	2.74	9.48	7.12	10.02	−25.61	1609.8	11.52
V02	Z1	13.52	8.21	6.27	8.77	−26.17	1319.6	16.56
V02	Z2	2.94	10.40	2.76	15.93	−17.83	1826.5	16.41
V03	Z1	−1.01	10.98	−3.49	17.98	−18.85	2152.4	12.37
V03	Z2	2.82	11.66	−0.86	18.10	−20.07	2061.5	9.27
V04	Z1	5.59	11.86	5.02	26.65	−14.63	1908.3	23.06
V04	Z2	−1.11	11.93	1.66	23.44	−16.32	1944.3	15.22
V05	Z1	−2.03	11.56	1.23	29.10	−13.17	2209.5	41.32
V05	Z2	−0.64	10.92	−2.94	22.59	−16.83	1948.6	27.94
V06	Z1	5.17	8.47	1.09	10.87	−23.88	1827.7	22.39
V06	Z2	2.89	10.31	0.33	13.66	−22.41	1689.8	17.93
V07	Z1	2.51	11.45	2.18	26.19	−12.79	1901.3	40.46
V07	Z2	5.90	11.05	5.80	18.99	−18.40	1591.7	20.68
V08	Z1	3.23	11.37	0.66	30.67	−11.63	2109.3	18.42
V08	Z2	6.29	10.03	8.36	18.83	−18.33	1790.5	40.06
V09	Z1	2.35	10.32	−3.15	8.87	−27.46	1518.6	32.45
V09	Z2	4.66	11.38	1.44	15.44	−20.62	1656.7	22.48
V10	Z1	0.93	10.48	−0.36	17.12	−21.82	2018.0	22.77
V10	Z2	−5.38	10.37	−4.51	14.93	−21.81	1965.7	11.65
V11	Z1	2.16	11.34	3.02	21.69	−16.95	1936.0	27.51
V11	Z2	1.81	11.86	−0.37	18.94	−19.60	1911.2	13.48
V12	Z1	4.72	10.28	2.70	14.10	−22.71	1625.3	13.53
V12	Z2	5.79	10.47	2.16	12.68	−23.52	1486.1	9.12
V13	Z1	0.67	11.30	−0.68	19.93	−19.11	1888.0	26.93
V13	Z2	1.98	10.64	−0.11	19.32	−18.44	1789.2	20.15

All values are means over valid analysis frames of the ‘tanto’ fragment, not frame-level or time-series quantities. Mean SFR and mean Q-value are whole-fragment averages of the same frame-level quantities used in the Stage-1 DTW analysis (Section 2.6) and are distinct from the DTW distance itself (Table 7). E03 mean Q-value at Z1 is not reported (0 valid frames).

The alpha ratio (energy ratio between 50–1,000 Hz and 1,000–5,000 Hz) reflects the balance between low- and high-frequency energy; lower values tend to indicate relatively richer high-frequency content. H1-H2 (the energy difference between the first and second harmonics, uncorrected for formant effects) is an acoustic correlate influenced by source-filter interaction and glottal configuration; it is not a direct closure measurement. The spectral centroid indexes the spectral center of gravity and is associated with timbral brightness. CPPS indexes the clarity of periodic structure and relative absence of noise. SPR represents the peak difference between the 2–4 kHz and 0–2 kHz regions and is a descriptive index of relative strength in the singer’s-formant-related region.

The 2.4–4.0 kHz analysis band used throughout this study is an operational, broadened window centered on the female singer’s-formant region reported by Bloothooft and Plomp (1986), who measured singer’s-formant level for female singers in a 1/3-octave band centered at 3.16 kHz. The present study’s 2.4–4.0 kHz band (approximately 3.16 ± 0.8 kHz) is the authors’ own operational widening of that reference band, adopted to accommodate individual differences, vowel, and pitch-dependent spectral variation; it is not itself derived from, or directly validated by, Bloothooft and Plomp (1986), and is referred to here as the singer’s-formant-related region rather than as a single fixed formant frequency. Because the target pitch, E♭5 (approximately 622 Hz), has a wide harmonic spacing, only a small number of harmonics fall within this band; Q-value, band-energy, and centroid estimates at this pitch are consequently sensitive to vowel quality, harmonic placement, and the linear-prediction procedure used to estimate the spectral envelope. This is discussed further in Limitations.

## 3 Results

### 3.1 Auditory-perceptual evaluation: descriptive overview

#### 3.1.1 Ratings and evaluative-term selections

Table 4 reports the individual T1-T4 ratings and their median for each participant. Median ratings ranged from 0 (E06, E07, V07, V08, V12) to 2 (V01, V03, V06). Because term selection was conditional on a non-zero individual rating (Section 2.5.2), the total number of selected terms is not treated here as an independent measure of development and is not correlated with the rating; it is reported (Table 6; full rater-by-term matrix in Supplementary Table S3) as descriptive information about how many distinct verbal characterizations accompanied a given participant’s evaluation, and it is used qualitatively in Section 3.3. Two participants illustrate that the 0-rating category is not terminologically uniform: E06 (median 0) received five term selections across raters (R02, R04, P05, R08, R07), and E07 (median 0) received four (R02 and S07 from three separate raters), indicating that raters heard and noted local change in these voices even though none judged the change to constitute overall bel canto development; by contrast, V07, V08, and V12 (all median 0) received zero term selections from any rater.

**Table 6:** Evaluative terms selected by each evaluator (T1-T4) for each participant, and the total number of selections (count; repeated selection across raters permitted; —, no term selected).

Subject	T1 terms	T2 terms	T3 terms	T4 terms	Total selections
E01	S01, R01, R02	S09, R04	R03, P05	R01, R04, R07, P02	11
E02	R06, R07, R09	R05, R09	S06, R03	S07, R06, R07, R09	11
E03	S04, R02, P02	R05	P01	R02, R05, R06	8
E04	R06	R05, P04	R07	R01, R02, R06, R07, P03	9
E05	S04, R06, R09	S10, R02, R06, P03	P05	R02, P04, P05	11
E06	R02, R04, P05	R08	—	R07	5
E07	R02	S07	S07	S07	4
V01	S04, R01, R09, P02, P05	S04, S06, R02, R05, P05	—	S04, R02, R09, P05	14
V02	S04, S10, R02, R06, P05	S04, S10, R02, R05	—	S04, R02, R05	12
V03	S09, S10, R01, R04, R06, R10	S09, R02, R04, R10, P05	S03, R06	S07, R02, R10, P05	17
V04	S05, S07, S09, R10	S05, S09, R08, P01, P03	—	—	9
V05	S02, S09, S10, R10	S02, S09, R10	—	—	7
V06	S04, S07, S09, R02, R04	S04, S09, R02, R04	P03	S04, R02, R04, R10	14
V07	—	—	—	—	0
V08	—	—	—	—	0
V09	R02, R05, R09, P02	S04, R05, R07	—	S04, S09, R02	10
V10	S09, R02, R06, R09	S09, R02, R06, R09	S03	R02, R06, R09	12
V11	S04, S09, R04	R06, R09	—	S09, R06, R09	8
V12	—	—	—	—	0
V13	R02, R06, R09	R10, P05	R06	S09, R09, R10	9

Term codes are defined in Table 3 (S, voice-source; R, resonance; P, respiratory/breath-support). This table is reproduced in full in Supplementary Table S3 with per-rater category tallies.

#### 3.1.2 Inter-rater agreement

Inter-rater agreement among the four evaluators’ 0–2 ratings was assessed using a two-way random-effects, absolute-agreement intraclass correlation coefficient (Koo and Li, 2016), computed on the raw 20 (participant) × 4 (rater) rating matrix. The average-measures coefficient was ICC(2,k) = 0.731 (bootstrap 95% CI, 5,000 resamples over participants: 0.49–0.85); the single-measures coefficient, relevant to the reliability of an individual rater, was ICC(2,1) = 0.404 (bootstrap 95% CI: 0.20–0.59). As a sensitivity analysis appropriate to the ordinal 0–2 scale, the mean pairwise quadratic-weighted kappa across the six rater pairs was 0.41 (range across pairs: 0.17–0.64), indicating that agreement was not uniform across rater pairs (T1-T3 and T2-T3 showed the lowest pairwise agreement). We report these values descriptively, as properties of this specific four-rater panel, rather than as evidence generalizable to voice pedagogues as a population, and we do not apply a categorical label such as ‘good agreement’ to a single point estimate with a wide confidence interval. This level and pattern of agreement is broadly consistent with prior research on expert assessment of musical performance. In a naturalistic study of 61 conservatoire recitals assessed by three experienced external evaluators, Thompson and Williamon (2003) reported a mean inter-evaluator correlation of ρ = 0.50 (range 0.33–0.65), accounting for only about a quarter of the observed variance, and reviewed further evidence that evaluators may arrive at holistic judgments through internalized, personal criteria that are difficult to express verbally and do not necessarily correspond to those of other judges. We therefore treat the moderate and rater-pair-dependent agreement observed here as a substantive feature of expert evaluation worth interpreting, rather than as a methodological defect specific to the present panel.

### 3.2 Stage 1: SFR/Q-value path-normalized DTW findings

Table 7 reports the path-normalized DTW distance from M2025 at Z1 and Z2, and the resulting Δproximity (positive = movement toward M2025), for SFR and for Q-value. The Q-value DTW distance could not be computed for E03 at Z1 because too few frames in that recording contained a detectable spectral peak in the analysis band (0 valid frames); this case is reported as ‘not computed’ rather than assigned an approach/divergence direction.

**Table 7:** Path-normalized DTW distance from M2025 (dimensionless) at Z1 and Z2 for SFR and Q-value, with Δproximity = DTWnorm(Z1) - DTWnorm(Z2).

Subject	Median	SFR DTWnorm Z1	SFR DTWnorm Z2	ΔSFR prox.	SFR direction	Q DTWnorm Z1	Q DTWnorm Z2	ΔQ prox.	Q direction
E01	1.5	2.83	2.78	0.05	Approach	15.42	11.44	3.98	Approach
E02	1.5	2.39	3.79	−1.40	Divergence	12.21	12.76	−0.55	Divergence
E03	0.5	5.09	2.70	2.39	Approach	—	23.40	—	Not computed
E04	0.5	2.96	2.89	0.07	Approach	19.21	13.88	5.32	Approach
E05	1.5	2.57	3.30	−0.73	Divergence	12.08	13.09	−1.01	Divergence
E06	0	4.76	2.90	1.86	Approach	24.23	13.74	10.49	Approach
E07	0	3.90	2.67	1.23	Approach	25.20	15.17	10.03	Approach
V01	2	2.72	2.99	−0.27	Divergence	14.89	14.99	−0.10	Divergence
V02	1.5	2.62	3.42	−0.80	Divergence	10.36	10.83	−0.47	Divergence
V03	2	2.50	3.10	−0.60	Divergence	11.13	14.41	−3.28	Divergence
V04	0.5	4.87	3.34	1.53	Approach	10.15	8.95	1.20	Approach
V05	0.5	5.94	4.01	1.94	Approach	12.10	11.25	0.85	Approach
V06	2	2.33	2.54	−0.21	Divergence	7.93	10.31	−2.38	Divergence
V07	0	4.55	3.19	1.36	Approach	11.05	9.36	1.69	Approach
V08	0	7.30	3.09	4.21	Approach	9.82	14.86	−5.05	Divergence
V09	1	2.35	2.52	−0.17	Divergence	14.42	10.64	3.78	Approach
V10	1.5	4.89	4.27	0.62	Approach	14.46	14.19	0.26	Approach
V11	1	3.75	3.62	0.13	Approach	9.59	12.10	−2.51	Divergence
V12	0	3.17	3.24	−0.07	Divergence	14.10	12.98	1.12	Approach
V13	1	3.28	2.77	0.50	Approach	10.82	10.26	0.57	Approach

Positive Δ indicates that Z2 was closer to M2025 than Z1 (‘approach’); negative Δ indicates ‘divergence’. Median development rating (from Table 4) is shown for reference. E03 Q-value at Z1 could not be computed (0 valid frames). DTWnorm = path-length-normalized dynamic time warping distance.

SFR-based distance decreased (approach) in 12 of 20 participants (60%) and increased (divergence) in 8 of 20 (40%). Q-value-based distance could be computed for 19 participants, of whom 11 (58%) showed approach and 8 (42%) showed divergence. Considering the 19 participants for whom both indices could be computed, both SFR and Q-value moved toward M2025 in 9 participants (E01, E04, E06, E07, V04, V05, V07, V10, V13); both moved away from M2025 in 6 participants (E02, E05, V01, V02, V03, V06); the two indices moved in opposite directions in the remaining 4 (SFR approach/Q divergence: V08, V11; SFR divergence/Q approach: V09, V12).

The correspondence between this acoustic movement and the median expert rating is, if anything, counter to a simple ‘closer to M2025 is better’ expectation. All six participants whose SFR and Q-value both moved away from M2025 (E02, E05, V01, V02, V03, V06) received high median ratings of 1.5 or 2. Conversely, among the nine participants whose SFR and Q-value both moved toward M2025, median ratings ranged widely, from 0 (E06, E07, V07) to 1.5 (E01, V10), with several intermediate values (E04 = 0.5, V04 = 0.5, V05 = 0.5, V13 = 1). SFR/Q-value proximity to M2025 therefore did not, in this sample, function as a proxy for perceived longitudinal development, and in some cases pointed in the opposite direction from the experts’ judgment. This descriptive pattern—rather than a formal predictive failure, which would require an inferential model this sample cannot support—motivates the case-level analysis in Section 3.3.

We do not interpret these directions as evidence that moving away from M2025 causes higher ratings, or that SFR/Q-value proximity is irrelevant; a plausible reading, developed further in Section 4, is that M2025 represents only one candidate reference point among several acoustic dimensions relevant to expert judgment, and that for participants already close to typical soprano configurations at Z1 (see V07 and V08 below), small further shifts toward or away from a single reference recording may carry little developmental meaning.

### 3.3 Stage 2: case-level correspondence between acoustic change and evaluative terms

Table 5 reports the whole-fragment (frame-averaged) values of the five additional acoustic measures, plus mean SFR and mean Q-value, at Z1 and Z2 for all 20 participants computed using the pipeline shown at Data availability statement. No regression or feature-importance model is fitted to these data; instead, we present a participant-level case matrix (Table 8) juxtaposing each participant’s median rating, SFR/Q-value DTW direction, acoustic-measure changes, and evaluative terms, and discuss selected contrasting cases in the text below.

**Table 8:** Participant-level case matrix combining the median development rating, SFR/Q-value DTW direction (Table 7), whole-fragment acoustic changes Z2 minus Z1 (Table 5), the union of evaluative terms selected by any rater (Table 6), and a brief interpretive note.

Subject	Median	SFR/Q DTW direction	ΔAlpha/ΔCPPS/ΔH1-H2/ΔCentroid/ΔSPR (dB/dB/dB/Hz/dB)	Evaluative terms selected (any rater)	Interpretive note
E01	1.5	appr./appr.	+2.0/−0.4/+5.0/−162/+0.9	S01, R01, R02, R04, R03, R07, P05, P02	Early-recording brightness/closure shift, resonance-oriented terms
E02	1.5	div./div.	−14.1/−1.3/−9.7/−357/−1.1	R06, R07, R09, R05, S06, R03, S07	Large decrease in alpha ratio and centroid despite divergence from M2025; brightness/closure terms
E03	0.5	appr./n.c.	−1.2/+1.2/−2.1/+827/+6.7	S04, R02, P02, P01, R05, R06	Mixed profile; Q-value not computable at Z1
E04	0.5	appr./appr.	0.0/+0.6/+5.1/+632/+4.5	R06, R05, P04, R07, R01, R02, R03	Approach on both indices but only moderate median rating
E05	1.5	div./div.	−5.5/+1.1/+8.5/+534/+6.6	S04, R06, R09, S10, R02, P03, P05	Divergence from M2025 with high median rating; resonance terms
E06	0	appr./appr.	−3.7/+1.9/−5.1/+970/+10.5	R02, R04, P05, R08, R07	Approach on both indices; local resonance/carrying-power terms but median 0
E07	0	appr./appr.	−3.8/+1.0/−2.4/+221/+3.5	R02, S07 (×3)	Approach on both indices; almost exclusively S07 (mouth-opening) and R02; median 0
V01	2	div./div.	−3.0/+0.6/+1.7/+73/+2.5	S04, R01, R09, P02, P05, S06, R05, R02	Divergence from M2025, richest resonance/breath term profile, median 2
V02	1.5	div./div.	−10.6/+2.2/−3.5/+507/+8.3	S04, S10, R02, R06, P05, R05	Divergence with high median rating
V03	2	div./div.	+3.8/+0.7/+2.6/−91/−1.2	S09, S10, R01, R04, R06, R10, S03, S07, P05	Divergence from M2025; broadest term set including depth (R10) and vibrato (S09), median 2
V04	0.5	appr./appr.	−6.7/+0.1/−3.4/+36/−1.7	S05, S07, S09, R10, R08, P01, P03	Approach on both indices; moderate median
V05	0.5	appr./appr.	+1.4/−0.6/−4.2/−261/−3.7	S02, S09, S10, R10	Approach on both indices; moderate median
V06	2	div./div.	−2.3/+1.8/−0.8/−138/+1.5	S04, S07, S09, R02, R04, P03, R10	Divergence from M2025; term set spans basic (S04, S07) and refined (S09, R10); median 2
V07	0	appr./appr.	+3.4/−0.4/+3.6/−310/−5.6	(none selected)	Approach on both indices; zero evaluative terms selected by any rater; median 0
V08	0	appr./div.	+3.1/−1.3/+7.7/−319/−6.7	(none selected)	SFR approach, Q divergence; zero evaluative terms; median 0
V09	1	div./appr.	+2.3/+1.1/+4.6/+138/+6.8	R02, R05, R09, P02, S04, R07, S09	Mixed direction indices; moderate median
V10	1.5	appr./appr.	−6.3/−0.1/−4.2/−52/0.0	S09, R02, R06, R09, S03	Approach on both indices; moderate-high median
V11	1	appr./div.	−0.4/+0.5/−3.4/−25/−2.7	S04, S09, R04, R06, R09	Mixed direction indices; moderate median
V12	0	div./appr.	+1.1/+0.2/−0.5/−139/−4.4	(none selected)	Mixed direction indices; zero evaluative terms; median 0
V13	1	appr./appr.	+1.3/−0.7/+0.6/−99/−6.8	R02, R06, R09, R10, P05, S09	Approach on both indices; moderate median

Appr., approach; div., divergence; n.c., not computed. This table is offered as descriptive, hypothesis-generating case material, not as a validated predictive account.

Several contrasts in Table 8 are informative for the interpretive framework developed in Section 3.4. First, the six participants with the highest median ratings and the clearest SFR/Q-value divergence from M2025 (E02, E05, V01, V02, V03, V06) show comparatively large decreases in alpha ratio (range −14.1 to −2.3 dB, with the exception of V03 at +3.8 dB) and, in several cases, increases in CPPS, co-occurring with resonance- and, for V01/V03/V06, depth- or vibrato-related terms (R09, R10, S09). This pattern is compatible with—though not proof of—a shift toward a brighter, more stable timbral quality that is not captured by SFR/Q-value proximity to a single reference recording.

Second, E06 and E07 illustrate that acoustic approach toward M2025 on both SFR and Q-value can co-occur with a median rating of zero. Both received their evaluative terms almost exclusively from the resonance category (R02, R04, R07, R08 for E06; R02 and, predominantly, S07 for E07), rather than the depth- or vibrato-related terms associated with high-median-rating participants. All four raters assigned E07 either R02 or S07, indicating that raters heard a local change (the mouth opening more adequately, or the voice sounding more resonant) without judging it sufficient to constitute overall bel canto development over the two-year period—consistent with a reading in which the expert focus for these two participants was on acquisition of basic vocal-production elements rather than on timbral/expressive refinement (see Section 4.2).

Third, V06 and V07—both members of the vocal-performance cohort—are a useful contrast pair precisely because cohort membership does not distinguish them: V06 received a median rating of 2, a large evaluative-term set spanning both basic (S04, S07) and refined (S09, R10) categories, and SFR/Q-value divergence from M2025; V07 received a median rating of 0 and zero evaluative-term selections from any rater, despite an SFR/Q-value profile that moved toward M2025 on both indices. Because raters did not know cohort membership, this difference cannot be attributed to an institutional label; it is more consistent with the raters treating V07’s Z1 recording as already comparatively established, such that no clear two-year change was perceived, and treating V06’s Z1 recording as containing an audible, resolvable limitation (this study’s methods cannot establish this directly; it is offered as a plausible, case-motivated reading, not a demonstrated finding). V08 and V12, which also received zero evaluative-term selections and a median rating of 0, together with V07 form a small set of cases in which the study’s acoustic and linguistic instruments jointly registered ‘no clear development,’ independent of the direction of SFR/Q-value movement.

These observations are presented as descriptive, case-based patterns motivating the interpretive framework in Section 3.4 and the discussion in Section 4; they are not the output of a fitted statistical model relating acoustic change to rating, and no claim is made that the acoustic changes reported here are the physiological cause, or a validated correlate, of the evaluative terms with which they co-occur.

### 3.4 Toward a Polanyi-centered interpretive framework

The case-level results above do not establish, and are not offered as establishing, a validated hierarchical or sequential cognitive structure. We therefore present a proposed, non-hierarchical interpretive framework (Figure 4). At its center is the focal awareness that pedagogically meaningful development has, or has not, occurred—corresponding to the 0–2 rating. Three sets of candidate contributors surround this focal judgment: (i) acoustic and auditory-perceptual particulars, of which SFR, Q-value, alpha ratio, H1-H2, spectral centroid, CPPS, and SPR are researcher-identified, *post hoc* candidates rather than evidence that experts consciously attend to these specific quantities; (ii) the 27 evaluative terms, understood after Vygotsky (1978) as cultural-linguistic tools that may allow experts to select, stabilize, and partially share elements of an otherwise holistic impression; and (iii) a developmental-pedagogical sensitivity, informed by Dreyfus and Dreyfus (1986), in which the same expert may attend to different qualities—basic vocal-function acquisition versus timbral/expressive refinement—depending on what is audible in a given recording, independent of the learner’s institutional cohort.

**Figure 4:**
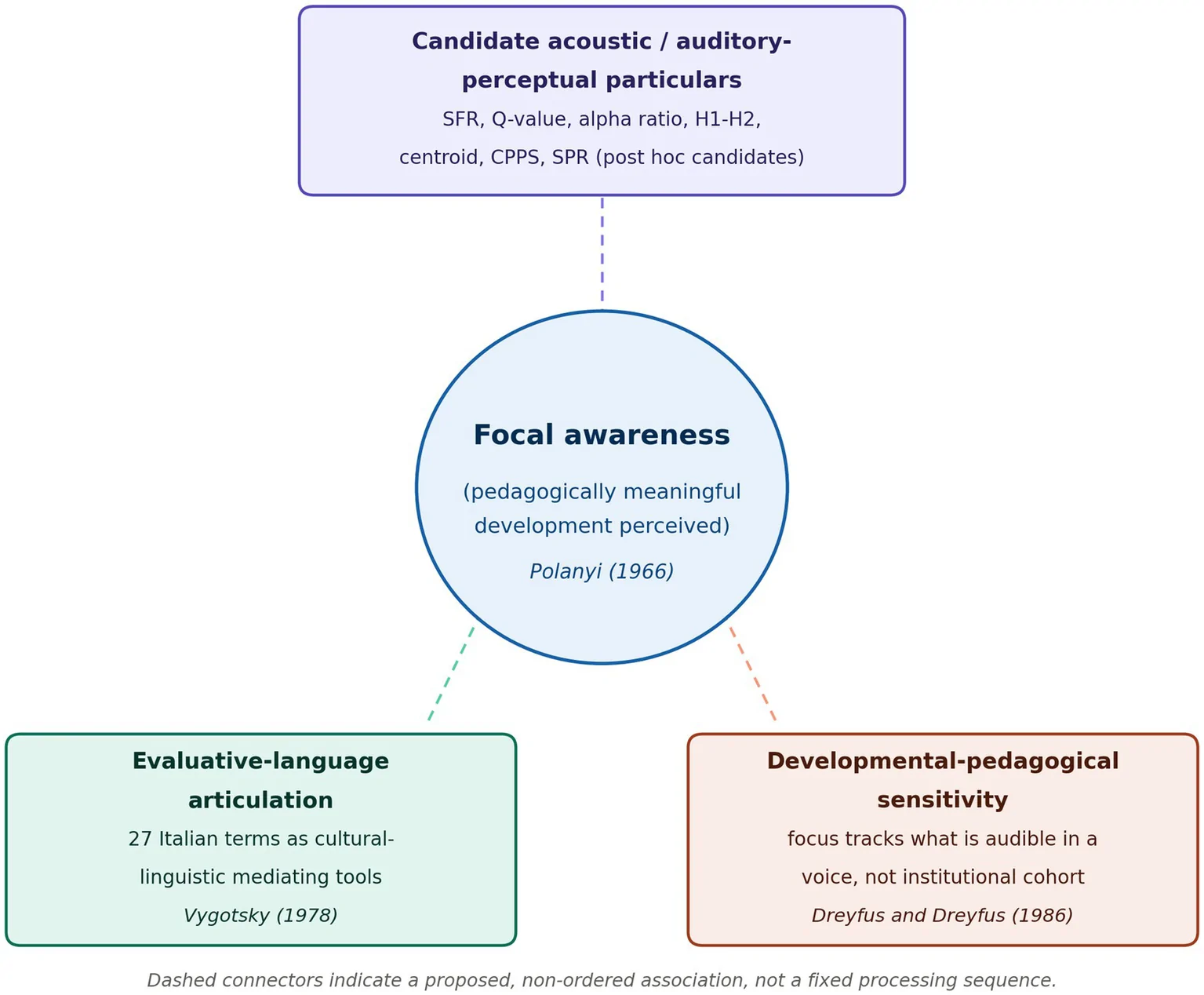
Proposed Polanyi-centered interpretive framework. Focal awareness (center) denotes the expert’s overall 0–2 judgment that pedagogically meaningful development has, or has not, occurred (Polanyi, 1966). Three sets of candidate, non-ordered contributors are shown: candidate acoustic/auditory-perceptual particulars (SFR, singer’s-formant ratio; CPPS, smoothed cepstral peak prominence; SPR, singing power ratio), evaluative-language articulation via the 27-item Italian vocabulary (Vygotsky, 1978), and developmental-pedagogical sensitivity to what is audible in a given voice, independent of institutional cohort (Dreyfus and Dreyfus, 1986). Dashed connectors denote a proposed association, not a demonstrated or ordered causal/processing sequence.

This framework is deliberately non-directional: we do not claim that acoustic particulars are processed first, then labeled with evaluative terms, then filtered through a developmental lens. All three elements are proposed as jointly, and only partially, related to the focal judgment, in the spirit of Polanyi’s claim that subsidiary particulars are integrated into a focal awareness that is not simply their sum. The framework is intended to generate specific, testable hypotheses for future, adequately powered and blinded studies (Section 6), not to be read as a confirmed cognitive model.

## 4 Discussion

This exploratory study examined candidate acoustic and linguistic correlates of expert tacit evaluative knowledge in bel canto pedagogy. Rather than asking which acoustic variables predict an expert rating, our analysis asks what the pattern of agreement and disagreement between SFR/Q-value proximity to a pedagogical reference recording and expert ratings, together with the evaluative terms selected in individual cases, can suggest about the kinds of information that may underlie expert judgment. The findings are consistent with—though they do not establish—a Polanyi-centered account in which no single acoustic index stands in for expert judgment.

### 4.1 SFR/Q-value proximity is not a proxy for perceived development

Contrary to a simple proximity-to-reference account, the six participants whose SFR and Q-value both moved away from M2025 over the 2 years received uniformly high median ratings (1.5–2), while several participants whose SFR and Q-value moved toward M2025 received low or zero median ratings (E06, E07, V07). This is a purely descriptive, case-level pattern from a sample of 20 non-randomly selected participants and four non-independent expert raters; it should not be read as evidence that moving away from a pedagogical reference model is developmentally desirable in general, nor as a demonstrated failure of SFR/Q-value measures. One plausible, non-exclusive reading is that M2025 is a single recording capturing one attainable configuration among several that experts may regard as pedagogically sound, so that departure from M2025’s specific SFR/Q-value profile does not necessarily indicate departure from bel canto principles more broadly.

### 4.2 Case-level evidence for multidimensional, term-mediated judgment

The case matrix (Table 8) suggests that experts’ evaluative-term choices track more than a single acoustic dimension. Participants receiving high median ratings and clear divergence from M2025 (e.g., V01, V03, V06) were associated with a broader range of terms spanning voice-source, resonance, and breath-support categories, including depth- and vibrato-related terms (R10, S09) not seen for the median-0 participants E06 and E07, whose terms were confined largely to mouth-opening and general resonance (S07, R02). We treat this as case-based, qualitative evidence consistent with Vygotsky's (1978) view of language as a tool that selects and stabilizes part of a holistic percept, rather than as evidence derived from a rating/term-count correlation.

### 4.3 Developmental sensitivity as a case-level, not cohort-level, lens

The V06/V07 contrast (Section 3.3) is, in our view, the clearest illustration in this dataset that expert evaluative focus can differ markedly between two students of the same institutional cohort, recorded and judged under the same blinding conditions. We read this as compatible with a Dreyfus-and-Dreyfus-informed interpretation in which the expert’s evaluative focus is sensitive to the developmental character of the problem currently audible in a given voice, rather than as confirmation that ‘developmental stage’ is a fixed, measurable property distinguishing the music-education and vocal-performance cohorts. This case-level view is also consistent with the broader expertise literature’s emphasis on the structured accumulation of individually tailored, feedback-guided practice—rather than cohort membership or elapsed calendar time alone—as the more proximal correlate of skill development. Ericsson et al. (1993), whose own empirical studies were conducted with music-academy violinists and pianists, define such deliberate practice as requiring tasks designed for the learner’s existing level together with immediate informative feedback, and argue that mere repetition or accumulated experience does not by itself yield maximal performance. This is compatible with why two vocal-performance-cohort students with the same nominal instructional exposure could nonetheless show very different two-year trajectories. Because these two cohorts differ in institution, curriculum, selection, and instructional exposure (Table 1), any cohort-level pattern in term usage is confounded with these factors and is reported, at most, as a preliminary qualitative observation rather than as a tested hypothesis about developmental stage as such.

### 4.4 Theoretical integration: Polanyi, Vygotsky, and Dreyfus and Dreyfus

We propose that these three theoretical resources are complementary rather than competing explanations. Polanyi's (1966) distinction between subsidiary and focal awareness supplies the theoretical center: acoustic measures such as alpha ratio, H1-H2, SPR, and CPPS are candidate correlates of subsidiary particulars that experts may not consciously analyze as separate cues, integrated instead into a holistic judgment that development has, or has not, occurred in a pedagogically meaningful way. Vygotsky's (1978) account of language as a psychological tool motivates treating the 27 evaluative terms as a culturally shared device for selecting, stabilizing, and communicating part of that holistic judgment, rather than as raw observational labels. Dreyfus and Dreyfus (1986) motivate treating the object of expert attention as sensitive to the developmental character of the specific problem audible in a voice, rather than to a learner’s institutional category.

This integration remains an interpretive proposal. No latent-variable, mediation, process-tracing, or model-comparison analysis in this study directly tests whether judgment proceeds through the three elements described above, in any order or combination; the theoretical framework should be evaluated on its capacity to generate further, more directly testable hypotheses (Section 6), not as a confirmed account of expert cognition.

### 4.5 Practical implications for singing-voice pedagogy

With the caveats above, the case analysis may still be practically useful. For example, the co-occurrence of decreased alpha ratio and increased CPPS with brightness- and resonance-related terms in several participants (Table 8) is compatible with, though not proof of, a link between these acoustic changes and a teacher’s judgment that ‘the voice resonates better’ or ‘the tone became brighter.’ Such correspondences, treated as hypotheses rather than established facts, may help teachers articulate part of the basis for an evaluation and may support learners’ self-monitoring, while the V06/V07 contrast illustrates that very different ratings can arise from qualitatively different underlying situations, cautioning against treating a single acoustic target as appropriate for all learners at a given nominal level.

### 4.6 Methodological contributions

Methodologically, this paper illustrates a discrepancy-driven, case-based strategy for studying tacit knowledge: rather than assuming that a single acoustic measure explains expert judgment, or fitting a statistical model that a small, non-random sample cannot support, the analysis first examines simple acoustic proximity to a reference recording (Stage 1), then investigates the cases in which proximity and expert judgment diverge, using additional acoustic measures and evaluative-term patterns considered case by case (Stage 2). We suggest this approach, together with explicit avoidance of underpowered inferential statistics, may be useful in other domains of embodied, language-mediated expertise.

## 5 Limitations

Several limitations qualify the interpretation of these results.

First, the sample comprised 20 participants (7 music-education, 13 vocal-performance) who were not randomly sampled and were not matched on prior training; statistical power for any inferential claim would be very low, which is why no regression, machine-learning, or between-group significance test is reported in this study. We recognize that a sample of this size, collected through an unusually labor-intensive two-year longitudinal protocol involving repeated recording, blinded expert listening, and multi-stage acoustic analysis, cannot support stable regression coefficients or generalizable importance rankings; this is why such models were not included rather than retained in weakened form. This sample size is nevertheless comparable to that of published longitudinal acoustic studies of singing-voice training, in which the per-participant cost of repeated, controlled recording over months or years similarly constrains cohort size: Mendes et al. (2003) followed 14 college voice majors across four consecutive semesters, and LeBorgne and Weinrich (2002) followed 21 first-year master’s-level vocal-performance majors across 9 months of training. This precedent contextualizes, but does not remove, the constraint that our own *N* = 20 places on statistical inference. The two cohorts differed in institution, curriculum, teacher, selection procedure, and instructional exposure (Table 1); any cohort-level difference is confounded with these factors and cannot be attributed to developmental stage alone.

Second, the four expert evaluators were not independent of the study materials: the same panel constructed the 27-item evaluative vocabulary, selected the M2025 reference recording, and rated all 20 participants. Vocabulary and reference-model selection preceded exposure to the Z1/Z2 acoustic and DTW results, which reduces, but does not eliminate, the risk of confirmation bias.

Third, the auditory-perceptual evaluation was not fully blinded: evaluators did not know participant identity or institutional cohort, and recordings were presented in randomized anonymized order, but each participant’s Z1 and Z2 recordings were presented as a temporally ordered pair (because the task was explicitly to judge longitudinal change), recordings were not loudness-normalized, and intra-rater (repeated-trial) reliability was not assessed. Inter-rater agreement was moderate to good depending on the index used (Section 3.1.2) and varied appreciably across rater pairs. Thompson and Williamon (2003) found that an evaluator’s own instrumental specialization could systematically shift the marks that evaluator awarded to performers on that instrument; the present panel comprised two sopranos, one mezzo-soprano, and one baritone, all judging female soprano voices, so an analogous influence of evaluator voice type cannot be excluded here. With four raters, the present design cannot test for such an effect, and we therefore note it as an uncontrolled source of rater variance rather than as a demonstrated bias.

Fourth, recording conditions used the built-in microphone and Auto gain setting of a single field recorder, without sound-pressure-level calibration or amplitude normalization; absolute loudness and inter-participant loudness comparisons are therefore not interpretable from these data. Room dimensions were documented for the vocal-performance cohort’s recording space but not for the music-education cohort’s; recording-condition consistency for each individual participant across Z1 and Z2 was maintained, but consistency between the two cohorts, and between the cohorts and M2025, is not fully documented.

Fifth, all analyses used the word ‘tanto’ as a single perceptual and acoustic unit, including consonantal onset and vowel transitions rather than an isolated steady-state vowel. This choice was motivated by the view that experts judge the word as an integrated whole (Section 1), but it means that measures such as H1-H2, Q-value, spectral centroid, and band-energy ratios were computed over frames that include transitional, non-steady-state acoustic content, which may increase variability in these estimates independent of any developmental change. The target pitch (E♭5, approximately 622 Hz) also produces widely spaced harmonics, so relatively few harmonics fall within the 2.4–4.0 kHz analysis band; Q-value, centroid, and band-energy estimates at this pitch are correspondingly sensitive to vowel quality and to the linear-prediction procedure used to estimate the spectral envelope (Section 2.7). H1-H2 was not corrected for formant effects and should be read as a source-filter-influenced acoustic correlate rather than a direct measure of glottal closure.

Sixth, M2025 is a single recording by a single professional singer, selected by the same non-independent expert panel, without independent external validation of its pedagogical appropriateness; it is presented, and should be read, as one attainable reference point among several that experts might regard as pedagogically sound, not as a unique or universal target.

Seventh, discrepancies between SFR/Q-value proximity and expert ratings, and between acoustic changes and evaluative-term selections, may reflect measurement error, recording-condition differences, rater variability, omitted acoustic dimensions, or the specific and narrow choice of M2025 as a single reference recording, in addition to—or instead of—genuine holistic or tacit aspects of expert judgment; we do not attribute unexplained variation automatically to tacit knowledge.

Eighth, generative AI tools (Generative AI statement) were used during manuscript preparation for translation, language editing, and draft code generation; all analysis code was reviewed by the authors, and results were checked against hand-calculated or spreadsheet-based examples for representative recordings and against deterministic re-computation of the full dataset, rather than relying on agreement between multiple AI systems as a form of independent validation.

## 6 Future directions

Future work should test specific, falsifiable hypotheses generated by the present framework—for example, whether decreased alpha ratio and increased CPPS reliably co-occur with brightness- and resonance-related evaluative terms in a larger, randomly sampled group of learners, evaluated by fully blinded and independent expert panels with repeated-trial reliability assessment. Studies with larger samples could support adequately powered regression or multilevel models, ideally pre-registered, together with cross-validation before any predictive claim is made. Independent construction and external pedagogical validation of reference recordings such as M2025, calibrated recording protocols, and stimulated-recall interviews with expert evaluators could further clarify how experts describe what they hear during real-time evaluation. Longitudinal research could also examine how learners internalize evaluative language as a tool for self-monitoring.

## 7 Conclusion

This exploratory, longitudinal study examined how four expert voice pedagogues evaluated singing-voice development in bel canto pedagogy from a brief fragment of a beginner’s singing. Movement of the singer’s-formant ratio and a Q-value index toward a pedagogical reference recording, M2025, computed using path-length-normalized dynamic time warping, did not correspond consistently with expert median ratings; strikingly, all six participants whose acoustic profile moved away from M2025 on both indices received high median ratings, while several participants who moved toward M2025 received low or zero ratings. Case-level comparison of five additional acoustic measures with the evaluative terms selected for individual participants suggests that experts attended to qualitatively different vocal qualities in different cases—basic vocal-production acquisition for some participants, timbral and expressive refinement for others—independent of institutional cohort, as illustrated by the V06/V07 contrast.

We propose a Polanyi-centered interpretive framework in which candidate acoustic particulars, the 27-item evaluative vocabulary (read through Vygotsky’s account of language as a cultural-cognitive tool), and a Dreyfus-and-Dreyfus-informed sensitivity to the developmental character of what is audible in a given voice are jointly, though only partially, related to experts’ focal judgment that pedagogically meaningful development has occurred. This framework is explicitly hypothesis-generating: given the small, non-random, non-independent-rater sample, no regression, machine-learning, or between-group inferential claim is made, and the framework is not presented as a validated cognitive structure. We hope the descriptive, case-based methodology illustrated here—examining discrepancies between simple acoustic proximity and expert judgment, then investigating those discrepancies with additional measures and evaluative language, at the level of individual cases—offers a template for studying tacit knowledge in other artistic and embodied domains of expertise.

## Data Availability

The datasets presented in this study can be found in online repositories. The names of the repository/repositories and accession number(s) can be found at: https://github.com/Soprano-Science/Program.

## References

[ref1] Bloothooft G. Plomp R. (1986). The sound level of the singer's formant in professional singing. J. Acoust. Soc. Am. 79, 2028–2033. doi: 10.1121/1.393211, 3722610

[ref2] Boersma P. Weenink D. (2025) Praat: doing phonetics by computer [computer program]. Version 6.4.27. Available online at: http://www.praat.org/ (Accessed September 30, 2026).

[ref3] Crisosto-Alarcón J. Maldonado-Delgado P. Rivas-Campos M. (2025). Conceptual metaphors about voice in the teacher-student interaction system during Western classical singing lessons. J. Voice. S0892-1997(25)00069-4. doi: 10.1016/j.jvoice.2025.02.021, 40055108

[ref4] Dreyfus H. L. Dreyfus S. E. (1986). Mind over Machine: The power of human Intuition and Expertise in the era of the Computer. New York, NY: Free Press.

[ref5] Ericsson K. A. Krampe R. T. Tesch-Römer C. (1993). The role of deliberate practice in the acquisition of expert performance. Psychol. Rev. 100, 363–406. doi: 10.1037/0033-295x.100.3.363

[ref6] Eyben F. Scherer K. R. Schuller B. W. Sundberg J. André E. Busso C. . (2016). The Geneva minimalistic acoustic parameter set (GeMAPS) for voice research and affective computing. IEEE Trans. Affect. Comput. 7, 190–202. doi: 10.1109/taffc.2015.2457417

[ref7] Fraile R. Godino-Llorente J. I. (2014). Cepstral peak prominence: a comprehensive analysis. Biomed. Signal Process. Control 14, 42–54. doi: 10.1016/j.bspc.2014.07.001

[ref8] Garellek M. Keating P. (2011). The acoustic consequences of phonation and tone interactions in Jalapa Mazatec. J. Int. Phon. Assoc. 41, 185–205. doi: 10.1017/s0025100311000193

[ref9] Hillenbrand J. Cleveland R. A. Erickson R. L. (1994). Acoustic correlates of breathy vocal quality. J. Speech Hear. Res. 37, 769–778. doi: 10.1044/jshr.3704.769, 7967562

[ref10] Koo T. K. Li M. Y. (2016). A guideline of selecting and reporting intraclass correlation coefficients for reliability research. J. Chiropr. Med. 15, 155–163. doi: 10.1016/j.jcm.2016.02.012, 27330520 PMC4913118

[ref11] LeBorgne W. D. Weinrich B. D. (2002). Phonetogram changes for trained singers over a nine-month period of vocal training. J. Voice 16, 37–43. doi: 10.1016/S0892-1997(02)00070-X, 12002885

[ref12] Liu J.-Y. Kamekawa T. Marui A. (2022). Acoustic expression of emotions in vocal performance: Vibrato variability in emotional singing styles. Acoust. Sci. Technol. 43, 201–204. doi: 10.1250/ast.43.201, 39974637

[ref13] McFee B. Raffel C. Liang D. Ellis D. P. W. McVicar M. Battenberg E. . (2015). librosa: audio and music signal analysis in Python. In Proceedings of the 14th Python in Science Conference (SciPy 2015), Austin, TX, 18–25. doi: 10.25080/Majora-7b98e3ed-003

[ref14] Mendes A. P. Rothman H. B. Sapienza C. Brown W. S. Jr. (2003). Effects of vocal training on the acoustic parameters of the singing voice. J. Voice 17, 529–543. doi: 10.1067/S0892-1997(03)00083-3, 14740934

[ref15] Omori K. Kacker A. Carroll L. M. Riley W. D. Blaugrund S. M. (1996). Singing power ratio: quantitative evaluation of singing voice quality. J. Voice 10, 228–235. doi: 10.1016/S0892-1997(96)80003-8, 8865093

[ref16] Patel S. Scherer K. R. Sundberg J. Bjorkner E. (2010). Acoustic markers of emotions based on voice physiology. In Proceedings of Speech Prosody 2010, Chicago, IL. International Speech Communication Association (ISCA). doi: 10.21437/speechprosody.2010-239

[ref17] Polanyi M. (1966). The Tacit Dimension. Garden City, NY: Doubleday.

[ref18] Sakoe H. Chiba S. (1978). Dynamic programming algorithm optimization for spoken word recognition. IEEE Trans. Acoust. Speech Signal Process. 26, 43–49. doi: 10.1109/tassp.1978.1163055

[ref19] Schubert E. Wolfe J. (2006). Does timbral brightness scale with frequency and spectral centroid? Acta Acust. United Ac. 92, 820–825. Available at: https://www.phys.unsw.edu.au/jw/reprints/SchubertWolfe06.pdf

[ref20] Sundberg J. (1974). Articulatory interpretation of the “singing formant”. J. Acoust. Soc. Am. 55, 838–844. doi: 10.1121/1.1914609, 4833080

[ref21] Sundberg J. (1987). The Science of the Singing Voice. DeKalb, IL: Northern Illinois University Press.

[ref22] Sundberg J. (2001). Level and center frequency of the singer's formant. J. Voice 15, 176–186. doi: 10.1016/S0892-1997(01)00019-4, 11411472

[ref23] Sundberg J. La F. M. Gill B. P. (2013). Formant tuning strategies in professional male opera singers. J. Voice 27, 278–288. doi: 10.1016/j.jvoice.2012.12.002, 23453594

[ref24] Sundberg J. Lindblom B. Hefele A. M. (2023). Voice source, formant frequencies and vocal tract shape in overtone singing: a case study. Logop. Phoniatr. Vocology 48, 75–87. doi: 10.1080/14015439.2021.1998607, 34860148

[ref25] Takahashi J. Toda N. Takemoto H. (2026). Vocal tract adjustments for expressing bright and dark timbres in operatic singing. Acoust. Sci. Technol. 47, 288–291. doi: 10.1250/ast.e25.90

[ref26] Thompson S. Williamon A. (2003). Evaluating evaluation: musical performance assessment as a research tool. Music. Percept. 21, 21–41. doi: 10.1525/mp.2003.21.1.21

[ref27] Vurma A. (2017). Phonatory strategies of male vocalists in singing diatonic scales with various dynamic shapings. J. Voice 31, 254.e17–254.e29. doi: 10.1016/j.jvoice.2016.06.018, 27469449

[ref28] Vygotsky L. S. (1978). Mind in Society: The Development of Higher Psychological Processes. Cambridge, MA: Harvard University Press.

[ref29] Wessel D. L. (1979). Timbre space as a musical control structure. Comput. Music. J. 3, 45–52. doi: 10.2307/3680283

[ref30] Yamashita Y. Kayama M. Ikeda K. Asanuma K. Itoh K. (2024). A study on acoustic analysis methods of frequency domain acoustic features related to proficiency of classical singing voice. IEEJ Trans. Electron. Inf. Syst. 144, 1197–1208. doi: 10.1541/ieejeiss.144.1197. (in Japanese)

[ref31] Yoshida S. Kayama M. Ikeda K. Yamashita Y. Yamaguchi M. Obata A. . (2020). Classical singing voice evaluation metrics based on acoustic features related to proficiency of vocal music. IEICE Trans. Inf. Syst. J103-D, 247–260. doi: 10.14923/transinfj.2019PDP0014. (in Japanese)

