# Supplementary material for: Toward a cognitive framework of expert singing-voice evaluation in bel canto pedagogy: a longitudinal exploratory study
Source: Front Psychol. 2026 Oct 7;17:1896993. doi: 10.3389/fpsyg.2026.1896993 (PMC13682987; doi:10.3389/fpsyg.2026.1896993)
Supplement: Supplementary file 1 [file Supplementary_file_1.docx]

**Supplementary Material**

*Toward a Cognitive Framework of Expert Singing-Voice Evaluation in Bel Canto Pedagogy: A Longitudinal Exploratory Study*

*Kyoko Ikeda, Michiko Yamaguchi, Akemi Obata, Tomohiro Tani, Yasuki Yamashita, Taisei Terauchi, Takashi Nagai, Yuko Mesuda, Mizue Kayama*

This document contains Supplementary Tables S1-S3 and the inventory of Supplementary Data Files S1-S7 referenced in Section 14 of the main article.

## Supplementary Table S1. Acoustic analysis parameters by measure

Frame size and window are common to all measures (2,048-sample analysis frames, Hann window, FFT size 2,048, 44.1 kHz sampling rate; Section 2.3.3 of the main article). The table below documents the measure-specific parameters and how frames that could not be reliably analyzed were handled. Full implementation is provided as source code in Supplementary Data Files S2 and S6.

*Note: 'Standard frame validity checks' refers to exclusion of frames containing digital silence or clipping, applied uniformly across measures; this does not involve imputation of missing analysis results. Exact hop size/overlap and LPC order are documented per script in the README files within Supplementary Data Files S2 and S6.*

| Measure | Frequency band / basis | Measure-specific parameters | Handling of unreliable frames |
| --- | --- | --- | --- |
| SFR (singer's-formant ratio) | 2.4-4.0 kHz energy relative to 0-4.0 kHz | RMS energy ratio per frame, expressed as a percentage | Standard frame validity checks only (silence/clipping) |
| Q-value | Spectral-envelope peak within 2.4-4.0 kHz | Peak center frequency divided by -3 dB bandwidth (fc/(fH-fL)); maximum Q within a frame used; LPC-based envelope estimation (order documented in Data S2) | Frames with no detectable peak in the band are excluded from that frame's Q-value series (not imputed, interpolated, or set to zero) |
| Alpha ratio | 50-1,000 Hz vs. 1,000-5,000 Hz | Energy-ratio index, dB | Standard frame validity checks only |
| H1-H2 (uncorrected) | First and second harmonic amplitude | Uncorrected for formant effects; harmonic amplitudes estimated from the frame spectrum | Frames without reliably identifiable H1/H2 peaks excluded from that frame's series |
| Spectral centroid | Full analysis band | Amplitude-weighted mean frequency, Hz | Standard frame validity checks only |
| CPPS | Full analysis band (cepstral domain) | Smoothed cepstral peak prominence, dB; smoothing window documented in Data S2 | Standard frame validity checks only |
| SPR (singing power ratio) | 2-4 kHz peak vs. 0-2 kHz peak | Peak-to-peak difference, dB | Standard frame validity checks only |

## Supplementary Table S2. Term-by-term interpretive notes (extends Table 3)

This table reproduces Table 3 of the main article together with the full interpretive note for each term, clarifying that the contextual English gloss is not a literal, clinical, or anatomical definition, and is not equated a priori with any single acoustic measure. Source data: Supplementary Data File S5.

| Code | Italian label | Contextual English gloss | Note |
| --- | --- | --- | --- |
| S01 | non forzare la voce | voice produced with less apparent forcing | Perceived/inferred reduction in forcing or tension; no direct physiological measurement is implied. |
| S02 | voce non ingolata (aperta) | less throaty or constricted, more open-sounding voice | Pedagogical contrast to a throaty/constricted sound; not an anatomical definition of an 'open throat'. |
| S03 | voce morbida | softer, more supple vocal quality | A perceptual/timbral descriptor; 'soft' here does not simply mean lower loudness. |
| S04 | voce non soffiata | less breathy, with reduced audible air leakage | Perceptual breathiness; not a direct measurement of airflow or glottal closure. |
| S05 | laringe abbassata (si ritiene) | production perceived as involving a lower laryngeal setting | Internal laryngeal action is perceived/inferred, not directly measured. |
| S06 | chiusura cordale | sound consistent with firmer vocal-fold closure and a stronger, fuller tone | Vocal-fold closure is inferred from the sound; it was not directly observed or measured. |
| S07 | la bocca si apre bene | more adequate and released mouth opening | The adequacy of mouth opening is perceived/inferred in the pedagogical context; it was not quantified. |
| S08 | intonazione giusta | more accurate intonation | Refers to perceived improvement in pitch accuracy. |
| S09 | vibrato naturale | more natural, well-formed vibrato | Perceptual evaluation of vibrato quality; not an operational definition of a vibrato metric. |
| S10 | giusta posizione delle vocali | more appropriate vowel placement and articulation | 'Position' is a pedagogical/articulatory concept, not a fixed anatomical coordinate. |
| R01 | sentire della risonanza | greater awareness or sensation of resonance | A pedagogical sensation/awareness of resonance; not a direct measurement of resonating cavities. |
| R02 | voce risonante | more resonant vocal quality | Perceptual resonance quality; not defined by a single acoustic variable. |
| R03 | si alza il palato molle | production perceived as involving a raised soft palate | Soft-palate action is perceived/inferred, not directly measured. |
| R04 | più voluminoso | greater vocal fullness or apparent volume | 'Voluminoso' can imply fullness as well as apparent volume; no calibrated SPL measurement is implied. |
| R05 | non è più chiusa dentro | less inward or muffled, more open-sounding voice | A pedagogical/perceptual description of reduced inward or muffled quality. |
| R06 | innalzamento del punto di risonanza | resonance perceived at a higher position | Resonance 'position' is a pedagogical percept or image, not a literal anatomical location. |
| R07 | voce in maschera | more forward, 'in-the-mask' resonance | Traditional bel canto term retained; the gloss indicates a perceived forward resonance, not a literal anatomical site. |
| R08 | voce che arriva lontano | voice with greater carrying power or projection | Perceived projection/carrying quality; it was not measured at a physical distance. |
| R09 | diventata più chiara | brighter vocal timbre | Perceptual brightness; not equated a priori with spectral centroid or any single acoustic measure. |
| R10 | voce profonda | greater depth and richness of vocal timbre | Perceptual timbral depth; not equated with lower pitch or a single spectral measure. |
| R11 | voce non nasale | less nasal vocal quality | Perceptual nasality; no nasalance measurement is implied. |
| R12 | unificazione delle cinque vocali | more unified and consistent resonance across the five vowels | Pedagogical consistency across vowels; does not imply that the vowels become acoustically identical. |
| P01 | respirazione costo-diaframmatica | breathing perceived as more effectively costo-diaphragmatic | Respiratory action is perceived/inferred; no direct respiratory measurement is implied. |
| P02 | sul fiato | voice carried on a well-coordinated breath flow | Traditional bel canto term retained; this is a contextual gloss of voice-breath coordination, not a literal or operational definition. |
| P03 | buon controllo del respiro | better control and management of the breath | Functional/pedagogical breath control; not a direct airflow measurement. |
| P04 | buon appoggio | more stable appoggio and breath-support coordination | Traditional bel canto term retained; the gloss does not reduce appoggio to a single physiological mechanism. |
| P05 | buon flusso del fiato | more continuous and well-coordinated breath flow | Perceived/functional breath flow; not a direct airflow measurement. |

## Supplementary Table S3. Complete rater-by-participant evaluative-term matrix

This table reproduces Table 4B of the main article in full, with the categories represented for each participant. — = no term selected by that evaluator. S = voice-source; R = resonance; P = respiratory/breath-support. Source data: Supplementary Data File S4.

| Subject | T1 terms | T2 terms | T3 terms | T4 terms | Total | Categories |
| --- | --- | --- | --- | --- | --- | --- |
| E01 | S01,R01,R02 | S09,R04 | R03,P05 | R01,R04,R07,P02 | 11 | P/R/S |
| E02 | R06,R07,R09 | R05,R09 | S06,R03 | S07,R06,R07,R09 | 11 | R/S |
| E03 | S04,R02,P02 | R05 | P01 | R02,R05,R06 | 8 | P/R/S |
| E04 | R06 | R05,P04 | R07 | R01,R02,R06,R07,P03 | 9 | P/R |
| E05 | S04,R06,R09 | S10,R02,R06,P03 | P05 | R02,P04,P05 | 11 | P/R/S |
| E06 | R02,R04,P05 | R08 | — | R07 | 5 | P/R |
| E07 | R02 | S07 | S07 | S07 | 4 | R/S |
| V01 | S04,R01,R09,P02,P05 | S04,S06,R02,R05,P05 | — | S04,R02,R09,P05 | 14 | P/R/S |
| V02 | S04,S10,R02,R06,P05 | S04,S10,R02,R05 | — | S04,R02,R05 | 12 | P/R/S |
| V03 | S09,S10,R01,R04,R06,R10 | S09,R02,R04,R10,P05 | S03,R06 | S07,R02,R10,P05 | 17 | P/R/S |
| V04 | S05,S07,S09,R10 | S05,S09,R08,P01,P03 | — | — | 9 | P/R/S |
| V05 | S02,S09,S10,R10 | S02,S09,R10 | — | — | 7 | R/S |
| V06 | S04,S07,S09,R02,R04 | S04,S09,R02,R04 | P03 | S04,R02,R04,R10 | 14 | P/R/S |
| V07 | — | — | — | — | 0 | None |
| V08 | — | — | — | — | 0 | None |
| V09 | R02,R05,R09,P02 | S04,R05,R07 | — | S04,S09,R02 | 10 | P/R/S |
| V10 | S09,R02,R06,R09 | S09,R02,R06,R09 | S03 | R02,R06,R09 | 12 | R/S |
| V11 | S04,S09,R04 | R06,R09 | — | S09,R06,R09 | 8 | R/S |
| V12 | — | — | — | — | 0 | None |
| V13 | R02,R06,R09 | R10,P05 | R06 | S09,R09,R10 | 9 | P/R/S |

## Supplementary Data Files S1-S7

The seven files below contain the complete primary data, analysis programs, and derived results underlying every number reported in the main article. ZIP file *Supplementary_Data_Files.zip* contains these files. Together they permit independent recomputation of Tables 2 and 4-7 and Figure 3 from the primary audio.

| File | Filename | Format | Content and relation to the article |
| --- | --- | --- | --- |
| Data S1 | Acoustic_Data_of_E01-E07-V01-V13-M2025.zip | ZIP (WAV) | Primary audio data: extracted 'tanto' fragments for all 20 participants at Z1 and Z2 (40 recordings) plus the M2025 pedagogical reference recording, as mono 44.1 kHz/16-bit linear PCM WAV (Section 2.3.1). Public sharing is covered by participant consent (Section 9). |
| Data S2 | Acoustic_Analysis_Programs.zip | ZIP (source code) | Analysis programs computing the seven acoustic measures (SFR, Q-value, alpha ratio, H1-H2, spectral centroid, CPPS, SPR), with per-measure parameter settings (Section 2.3.3; Supplementary Table S1). |
| Data S3 | Acoustic_Analysis_Results_2026-08-18.zip | ZIP (CSV) | Frame-level and whole-fragment acoustic output for all participants and M2025, including the consolidated matrix from which Tables 2 and 6 were computed. |
| Data S4 | Auditory_Evaluation_Summary_by_Four_Professionals.xlsx | XLSX | De-identified rater-level evaluation data: individual T1-T4 development scores, rater-wise medians, and evaluative-term selections (Tables 4A, 4B; Supplementary Table S3). |
| Data S5 | BelCanto_Evaluative_Terms_4cols_Code_Italian_EnglishGloss_Note.xlsx | XLSX | The 27-item Italian evaluative vocabulary with term codes, contextual English glosses, and full interpretive notes (Table 3; Supplementary Table S2). |
| Data S6 | DTW_SFR_Q_GitHub_Code_README_2026-08-18.zip | ZIP (source code) | Path-length-normalized DTW implementation and README, documenting the local cost function, permitted step directions, and normalization procedure (Section 2.6). |
| Data S7 | DTW_SFR_Q_Path_Aligned_CSV_and_Plots_2026-08-18.zip | ZIP (CSV, PNG) | Path-aligned DTW output: per-participant normalized distances at Z1 and Z2 with cumulative cost and path length (Table 5), aligned CSV series, and the alignment plots from which Figure 3 was produced. |

*The analysis code in Data S2 and Data S6 is additionally mirrored at https://github.com/Soprano-Science/Program.*
